# Supplementary material for: Correlation between obesity and clinicopathological characteristics in patients with papillary thyroid cancer: a study of 1579 cases: a retrospective study
Source: PeerJ. 2020 Sep 8;8:e9675. doi: 10.7717/peerj.9675 (PMC7485482; doi:10.7717/peerj.9675)
Supplement: Supplemental Information 1 [file peerj-08-9675-s001.docx]

S1. Logistic regression of BMI level on different adverse clinico-pathological characteristics (**male**)

|  | BMI<18.5  N=0 | 18.5≤BMI＜24  N=78 | 24≤BMI＜28  N=141 | BMI≥28  N=127 |
| --- | --- | --- | --- | --- |
| Multifocality | | | | |
| OR(95%CI) | — | Reference | 1.35(0.74,2.44) | 1.16(0.63,2.13) |
| P | — |  | 0.33 | 0.63 |
| Tumor size≥ 1 cm | | | | |
| OR(95%CI) | — | Reference | 1.15(0.66,2.03) | 1.26(0.71,2.22) |
| P | — |  | 0.62 | 0.43 |
| Extrathyroidl extension | | | | |
| OR(95%CI) | — | Reference | 1.43(0.66,3.10) | 0.79(0.34,1.86) |
| P | — |  | 0.37 | 0.59 |
| lymph node metastasis | | | | |
| OR(95%CI) | — | Reference | 0.73(0.42,1.29) | 0.63(0.35,1.11) |
| P | — |  | 0.28 | 0.11 |
| T staging | | | | |
| OR(95%CI) | — | Reference | 1.50(0.69,3.24) | 0.79(0.34,1.86) |
| P | — |  | 0.30 | 0.59 |
| TNM staging (stage III + IV) | | | | |
| OR(95%CI) | — | Reference | 3.19(0.83,12.23) | 0.87(0.17,4.31) |
| P | — |  | 0.09 | 0.86 |
